# Negative Impact of Smaller Hometown Size on the Educational Experience of Medical Students: A Nationwide Study in Saudi Arabia

**DOI:** 10.7759/cureus.60342

**Published:** 2024-05-15

**Authors:** Hussain M Alkhars, Abdullah Alkhars, Ahmed M Al-Tayeb, Mohammed Aleid, Abdullah AlKarni, Moath Alowairdhi, Afaf Altayeb, Faisal H Abed, Mohammed Alessa

**Affiliations:** 1 Medicine, School of Medicine and Health Sciences, George Washington University, Washington, DC, USA; 2 Pediatrics, King Faisal University, Al-Ahsa, SAU; 3 Emergency Medicine, School of Medicine and Health Sciences, George Washington University, Washington, DC, USA; 4 General Surgery, University of Pennsylvania Health System, Philadelphia, USA; 5 Otolaryngology Head and Neck Surgery, King Saud Bin Abdulaziz University for Health Sciences College of Medicine, Riyadh, SAU; 6 Medicne, College of Medicine Alfaisal University, Riyadh, SAU; 7 Medicne, College of Medicine, Imam Abdulrahman Bin Faisal University, Dammam, SAU; 8 General Surgery, King Faisal University, Al-Ahsa, SAU

**Keywords:** clinical medical education, gender disparities, hometown size, saudi arabia, specialty exposure

## Abstract

Background and objective

Saudi Arabia's rapid medical education expansion has posed unique challenges for its students, particularly concerning specialty selection. Having broad exposure to medical specialties is crucial for making informed decisions. This study explores how the size of students' hometowns influences their exposure to their preferred specialty, thereby affecting their choice.

Methods

Our cross-sectional study collected data from medical students in their 4th and 5th years, interns, and graduates across Saudi Arabia. An electronic survey gathered information about medical specialty choice, interest levels, students’ self-ranking compared to their peers, and level of exposure to the chosen specialty. Overall exposure to specialties was quantified by tallying participants' experiences in preclinical observerships, didactic lectures, research projects, core and elective rotations, and attended conferences. We divided the students into three city sizes: primary urban centers, intermediate urban cities, and small townships and compared the outcomes between these three groups.

Results

Responses were obtained from 1,072 participants, with 424 (39.6%) from primary urban centers, 367 (34.2%) from intermediate urban cities, and 281 (26.2%) from small townships. Student hometown size was an independent predictor of specialty exposure, with students from smaller cities reporting lower exposure scores (OR = 0.73, (0.63-0.84), p<0.01). The study also identified gender disparities in exposure, with female students found to be correlated with a lower exposure score (OR = 0.72, (0.58-0.89), p<0.01).

Conclusion

City size is a significant determinant of specialty exposure for Saudi medical students. These findings highlight the need for initiatives that promote equal educational experiences, ensuring comprehensive specialty exposure to all students.

## Introduction

The inception of medical education in Saudi Arabia dates back to 1967, with King Saud University offering the first medical degree. Initially, medical training was limited to a few cities, accommodating only a small number of students. However, the turn of the century marked a period of rapid growth, with 18 new medical schools established across 11 cities between 2001 and 2008, a stark contrast to the gradual evolution of medical education systems seen internationally [1].

This rapid growth has posed challenges for Saudi medical students, many of whom are set to become the first generation of physicians in their families and cities. The lag in developing educational infrastructure to match medical service needs has led many Saudi physicians to train abroad. Moreover, a reliance on expatriate professionals persists, with non-citizens constituting 67.2% of the physician workforce in 2018, underscoring the country's ongoing physician shortage in several specialties [2].

As the number of medical colleges in Saudi Arabia grows, so does the complexity of specialty selection for medical students. Previous studies have generally been conducted in single institutions in the country’s largest cities, although Saudi Arabia encompasses 13 distinct regions [3-8]. This study aims to address this gap by examining students from across the country.

The level of exposure to medical specialties has been identified as a significant factor influencing students' specialty preferences, which is the central focus of our research [9, 10]. Specifically, we studied how the size of the students’ hometown affects their exposure to their chosen specialty. We anticipated that the varied distribution of teaching hospitals and resources across the different geographical regions within the country would affect students' experiences, with students from smaller cities having less exposure to their chosen specialty [11]. The findings aim to inform efforts to develop a balanced healthcare workforce attuned to the country's unique needs.

## Materials and methods

Study design and setting

This prospective cross-sectional study, conducted during the years 2020-2021, collected responses from medical students from various cities across the Kingdom of Saudi Arabia. To ensure a representative sample, recruitment targeted 40-50% of participants from primary urban centers, 30-40% from intermediate urban cities, and 20-30% from smaller cities. Participants included 4th-year and 5th-year medical students, interns, and graduates yet to match into a training program from any Saudi medical school.

The students’ hometown sizes were classified into three distinct urban settings: 'Primary urban centers' included major metropolises such as Riyadh, Dammam, and Jeddah, characterized by significant population densities and robust economic activities. 'Intermediate urban cities' encompassed moderately populated areas with developing economic functions, such as Mecca, Medina, Taif, Qassim, and Al-Ahsa. Lastly, 'small townships' included remote cities with smaller populations and limited economic diversification, such as Turaif, Sakaka, Najran, and Jazan.

This study received ethical approval from the Institutional Review Board (IRB) of King Fahad Medical City (approval number: IRB00010471). Participants electronically consented to contribute anonymized data upon starting the online survey. Recruitment was achieved through electronic communication coordinated by school representatives, with follow-up reminders ensuring the predefined distribution of respondents was met.

Questionnaire

The questionnaire was developed in three steps: 1) a comprehensive literature review was conducted, and the identified questionnaires were assessed by the authors; 2) items relevant to the study were adopted, and new items were added to address the study's objectives; 3) a pilot study on 15 students was conducted and edits were made accordingly. The final version included four sections: demographic data, academic data, personal interest, and exposure to the field. The personal interest section included a question to indicate the preferred specialty, the degree of interest, and the number of times they have changed their specialty preference. Exposure to the field was assessed by tallying the participants' preclinical observerships, didactic lectures, research experience, core rotations, elective rotations, and attendance at national or international conferences. Survey items assessing exposure were graded appropriately in a multiple-choice format. Items assessing participants’ interest used a sliding scale that ranged from 0-100. The questionnaire was administered virtually using the web-based service REDcap, and all questions were voluntarily and anonymously answered [12, 13].

Data analysis

Data management and analysis were carried out using REDCap hosted at Children’s National Hospital [12, 13]. Research Electronic Data Capture (REDCap) is a secure, web-based software platform designed to support data capture for research studies, providing comprehensive audit trails, automated exports to statistical packages, and robust data integration features. Throughout the study period, the completion of questionnaires was routinely monitored, with only fully completed surveys included in the analysis. Recruitment continued until enough complete responses were received to fulfill the recruitment targets.

Descriptive statistics were used alongside Pearson’s coefficient and the Student's t-test to assess binary and continuous variables, respectively. Multivariate ordinal regression analysis was then applied to explore the impact of grade point average (GPA), personal interest, self-ranking, and hometown size on exposure to selected specialties. Significance was determined at a p-value of less than 0.05, with analyses conducted using IBM SPSS Statistics v. 28.

## Results

Demographic and academic characteristics of surveyed medical students

Responses from 1072 medical students in their clinical, intern, and service years were collected. 536 (50.0%) of the medical students were females with a mean age ± SD of 24.0 ± 2.3 years. Students reported interest in a wide range of medical specialties listed in Table 1. 

**Table 1:** Demographic and academic characteristics of participating medical students.

Characteristic	Value
Age, mean (SD)	24.0 (2.3)
Female, N (%)	536 (50.0)
City, N (%)	-
Primary urban center	424 (39.6)
Intermediate urban city	367 (34.2)
Small township	281 (26.2)
Year in medical school, N (%)	-
Fourth year	147 (13.7)
Fifth year	458 (42.7)
Intern year	392 (36.6)
Graduate/Service	75 (7.0)
Chosen specialty	-
Internal Medicine	347 (32.4)
General Surgery	207 (19.3)
Pediatrics	200 (18.7)
Emergency Medicine	165 (15.4)
Family Medicine	227 (21.2)
OB/GYN	90 (8.4)
Anesthesiology	78 (7.3)
Radiology	113 (10.5)
Psychiatry	98 (9.1)
Neurology	80 (7.5)
Ophthalmology	105 (9.8)
Dermatology	79 (7.4)
Orthopedic Surgery	108 (10.1)
Otorhinolaryngology	91 (8.5)
Neurological Surgery	60 (5.6)

Medical students GPA, self-assessment, and level of interest 

Students from primary urban centers and intermediate urban cities had a higher GPA compared to students from small townships (primary vs. small: p<0.01; intermediate vs. small: p<0.01) (Table 2). When asked to rank their performance compared to their peers, students from primary urban centers ranked their performance at the 70th percentile, significantly higher than students from small townships who ranked themselves at the 64th percentile (p<0.01). Despite achieving a higher GPA, students from intermediate urban cities also ranked their performance lower than those from primary urban centers, at the 65th percentile (intermediate vs. primary: p<0.01; intermediate vs. small: p=0.72). Furthermore, students from primary urban centers reported the highest interest in their specialty (primary vs. intermediate: p<0.01; primary vs. small: p<0.01; intermediate vs. small: p=0.58). Interestingly, students from small townships changed their chosen specialty more frequently than students from primary urban centers (p<0.01).

**Table 2:** Medical students' GPA, self-rank, specialty interest, and frequency of specialty reconsideration divided by student hometown size. GPA: Grade Point Average.

Factor	Primary urban center	Intermediate urban city	Small township	P-value		
				Primary vs Intermediate	Primary vs Small	Intermediate vs Small
GPA, mean (SD)	4.2 (0.5)	4.3 (0.5)	4.1 (0.6)	0.82	<0.01	<0.01
Self-ranked academic standing, mean (SD)	69.8 (20.3)	65.3 (20.0)	64.2 (19.3)	<0.01	<0.01	0.72
Personal interest in the specialty, mean (SD)	82.3 (17.8)	76.2 (17.7)	77.7 (19.3)	<0.01	<0.01	0.58
Specialty change frequency, N (%)	-	-	-	-	-	-
Never	92 (21.7)	65 (17.7)	39 (13.9)	0.18	0.011	0.23
1-2 times	219 (51.8)	194 (52.9)	140 (49.8)	0.82	0.12	0.20
3-4 times	112 (26.5)	108 (29.4)	102 (36.3)	0.40	<0.01	0.077

Specialty exposure levels based on the students’ hometown size

Overall exposure was quantified through preclinical observerships, didactic lectures, research projects, core and elective rotations, and conferences attended, culminating in an overall exposure score (Table 3). Students from primary urban centers reported the highest exposure score of 7.87 ± 3.56, significantly higher than those from intermediate urban cities at 7.05 ± 3.60 and small townships at 6.36 ± 3.95 (primary vs. intermediate: p<0.01; primary vs. small: p<0.01).

**Table 3:** Saudi medical students’ exposure to chosen specialty via various educational activities across city size.

Factor	Primary urban center	Intermediate urban city	Small township	P-value		
				Primary vs Intermediate	Primary vs Small	Intermediate vs Small
Preclinical observerships, N (%)	-	-	-	0.86	0.019	0.017
None	103 (24.3)	94 (25.6)	83 (29.6)	-	-	-
40< hours +1	210 (49.5)	169 (46.0)	144 (51.4)	-	-	-
40-80 hours +2	71 (16.7)	66 (18.0)	36 (12.9)	-	-	-
>80 +3	40 (9.4)	38 (10.4)	17 (6.1)	-	-	-
Preclinical didactic lectures, N (%)	-	-	-	0.19	0.019	<0.01
None	77 (18.2)	56 (15.3)	59 (21.0)	-	-	-
0-10 hours +1	157 (37.0)	128 (34.9)	127 (45.2)	-	-	-
10-30 hours +2	132 (31.1)	132 (36.0)	62 (22.1)	-	-	-
>30 hours +3	58 (13.7)	51 (13.9)	33 (11.7)	-	-	-
Research projects, N (%)	-	-	-	0.47	0.98	0.53
None	136 (32.2)	132 (36.0)	90 (32.0)	-	-	-
1 project +1	136 (32.2)	112 (30.5)	89 (31.7)	-	-	-
2 projects +2	91 (21.5)	63 (17.2)	66 (23.5)	-	-	-
3 or more projects +3	60 (14.2)	60 (16.3)	36 (12.8)	-	-	-
Core rotations, N (%)	-	-	-	<0.01	<0.01	0.063
None	77 (18.2)	107 (29.2)	121 (43.4)	-	-	-
<2 weeks +1	66 (15.6)	79 (21.6)	39 (14.0)	-	-	-
2-4 weeks +2	117 (27.7)	96 (26.2)	43 (15.4)	-	-	-
>4 weeks +3	163 (38.5)	84 (23.0)	76 (27.2)	-	-	-
Elective rotations, N (%)	-	-	-	<0.01	<0.01	0.66
None	130 (30.7)	142 (38.8)	126 (45.0)	-	-	-
<2 weeks +1	42 (9.9)	57 (15.6)	24 (8.6)	-	-	-
2-4 weeks +2	116 (27.4)	99 (27.0)	71 (25.4)	-	-	-
>4 weeks +3	135 (31.9)	68 (18.6)	59 (21.1)	-	-	-
Conferences attended, N (%)	-	-	-	0.28	<0.01	0.064
None	213 (50.2)	192 (52.3)	167 (59.4)	-	-	-
National conference +1	147 (34.7)	135 (36.8)	90 (32.0)	-	-	-
International conference +2	37 (8.7)	27 (7.4)	18 (6.4)	-	-	-
National and international conferences +3	27 (6.4)	13 (3.5)	6 (2.1)	-	-	-
Overall score, mean (SD)	7.87 (3.56)	7.05 (3.60)	6.36 (3.95)	<0.01	<0.01	0.059

When examining specific educational components, students from small townships attended shorter preclinical observerships compared to those from primary urban centers (p=0.019) and intermediate urban cities (p=0.017). A similar pattern was observed for preclinical didactic lectures, where a significantly higher percentage of students from small townships attended fewer lectures than their peers (primary vs. small: p=0.019; intermediate vs. small: p<0.01).

Additionally, a larger proportion of students from primary urban centers participated in longer core rotations (primary vs. intermediate: p<0.01; primary vs. small: p<0.01) and elective rotations (primary vs. intermediate: p<0.01; primary vs. small: p<0.01).

Regarding extracurricular activities, a larger percentage of students from primary urban centers attended more national and/or international conferences compared to those from small townships (p<0.01) but not those from intermediate urban cities (p=0.28). Students' research experiences were consistent across city sizes with no significant difference in the number of research projects reported (primary vs intermediate p=0.98, primary vs small p=0.47, intermediate vs small p=0.53).

Factors influencing specialty exposure in medical students

Our multivariate ordinal regression analysis revealed that hometown size was a significant predictor, with students from smaller cities reporting lower exposure scores than their counterparts (OR = 0.73, [0.63-0.84], p<0.01) (Table 4). Students with a strong personal interest in their specialty were more likely to report higher levels of exposure (OR = 1.02, 95% CI (1.01-1.03), p<0.01). Similarly, self-ranked academic standing was positively correlated with exposure score (OR = 1.02, (1.01-1.02), p<0.01).

**Table 4:** Multivariate analysis of the negative influence of less urbanized hometowns on the exposure score of medical students in Saudi Arabia. GPA: Grade Point Average.

Variable	OR (CI 95%)	P-value
Less urbanized hometown	0.73 (0.63−0.83)	<0.01
GPA	0.71 (0.57−0.88)	<0.01
Personal interest in the specialty	1.02 (1.01−1.03)	<0.01
Self-ranked academic standing	1.02 (1.01−1.02)	<0.01
Female	0.72 (0.58−0.89)	<0.01

Contrary to expectations, a higher GPA was associated with a lower likelihood of reporting a high exposure score (OR = 0.70, (0.57-0.88), p<0.01). Gender was another significant negative predictor; female students were correlated with lower exposure scores (OR = 0.72, (0.58-0.89), p<0.01).

## Discussion

Broad exposure to medical specialties is key for medical students to make well-informed career decisions [9, 14, 15]. Our study, which encompassed students from three urban settings, namely, primary urban centers, intermediate cities, and small townships, revealed a clear link between the size of the students’ hometown and their exposure to their chosen specialty. Exposure was highest for students from primary urban centers, followed by those from intermediate urban cities, and lowest for students from small townships. Importantly, we found that students with lower exposure scores were more likely to report lower interest in their chosen specialty and were more likely to perceive their academic standing as lower compared to their peers.

Clinical exposure is increasingly recognized for its educational value, with many advocating for preclinical observerships as a form of early clinical exposure [16]. In line with this, more than 70% of students in our study reported engaging in preclinical observerships across the city sizes. The importance of preclinical observerships is substantial for specialties such as radiology, anesthesiology, and surgery, which students often have minimal exposure to prior to their clinical years. These experiences, when delivered through structured programs with active engagement from faculty, have been found to decrease the students' apprehension about entering these specialties [17]. In fact, studies examining preclinical surgical observerships found that experiences as short as one day can increase students’ interest and improve their perception of the specialty [18]. It is encouraging to find that the majority of our study's participants have pursued preclinical observerships. Notably, despite students from smaller townships having shorter observerships, this does not diminish the early understanding gained from these experiences.

Clinical rotations have a larger influence on students’ career choices. They are designed to be immersive experiences, where students get to experience working as part of the service team [19]. Previous studies have revealed that clinical clerkships affect students in distinct ways. Firstly, for students who had a predetermined specialty choice, the clerkships reinforce their choice and increase their interest [20]. Secondly, for those who end up changing their chosen specialty, positive encounters with dedicated educators can prompt them to switch to previously unconsidered specialties. Unfortunately, the opposite is also true as negative experiences have been found to deter students from their initial choices [19, 21]. In our study, a higher percentage of students from major urban centers reported participating in longer core and elective rotations compared to their counterparts from small townships. We hypothesize that the limited exposure to their chosen specialties in small townships may contribute to these students' uncertainty, as reflected by the frequent reconsideration of their specialty choice. Without an engaging clinical experience, students might fail to appreciate the scope and nature of each specialty, limiting their ability to commit.

Didactic lectures and conferences are other avenues for students to explore different medical specialties [22-25]. Students in primary urban centers benefit from a broader range of lectures due to the presence of more diverse faculty members in their schools compared to those from small townships. Encouraging students to attend professional meetings will allow them to explore their desired specialty and engage with role models [26]. Active promotion of these meetings to students from small townships is especially important, given that they reported significantly lower attendance than students from primary urban centers. This could be addressed by providing travel awards to interested students either through medical schools or the specialty’s national organizations as an incentive.

Our analysis investigated whether personal characteristics might affect the observed link between students' exposure to medical specialties and the size of their home cities. The multinomial ordinal regression revealed that home city size remains a significant predictor of exposure level, even after adjusting for GPA, personal interest, gender, and student’s self-rank. This suggests that the disparity in exposure reflects a broader issue affecting all students who come from less urbanized towns. This risk perpetuates the existing uneven geographical distribution of medical professionals around the country [11]. Students from these less urbanized cities are crucial to addressing the existing shortage in their communities, but they seem to encounter significant challenges in accessing training opportunities. This calls for a concerted effort to address these challenges to work towards equitable healthcare distribution nationwide.

Female gender was an independent predictor of lower exposure scores, suggesting that women are disproportionately affected by the uneven distribution of educational opportunities. This could be a result of societal norms limiting females’ ability to travel. Furthermore, with women representing only 38% of physicians in Saudi Arabia and even fewer in consultant roles, the scarcity of female role models in some specialties could decrease female students’ engagement in fields with less female representation [27-29]. This imbalance is anticipated to diminish as more female physicians complete their training under the expanding healthcare system. Nonetheless, proactive mentorship for female medical students is key and should be endorsed by medical colleges and teaching hospitals to promote equitable educational experiences.

The disparity in educational opportunities has major implications. We found that decreased exposure scores were correlated with both reduced interest in chosen specialties and lower confidence in their abilities, as indicated by their self-assessment compared to peers. In studies of work psychology, positive self-evaluation and intrinsic motivation are vital and can predict job engagement and satisfaction [30]. Certainly, medical training can be arduous, and a strong sense of commitment is needed for the trainees to thrive during residency. Therefore, it is paramount for stakeholders such as the Ministry of Health, teaching hospital centers, and medical colleges to improve the educational experience for students, especially those from less urbanized cities. This will enable a consistent supply of well-trained medical professionals to areas with the greatest needs, addressing critical healthcare shortages.

Strengths and limitations of the study

To our knowledge, this is one of the first studies to recruit a large sample of medical students from multiple institutions nationwide. This allowed us to identify significant associations that previous single-institution studies failed to reveal. Our findings can serve as a steppingstone for future policies to ensure equitable access to educational opportunities.

Nevertheless, our study suffers from several limitations. The cross-sectional design, while efficient and appropriate for the purpose of our study, does not assess how the effects of the factors studied change over time. Additionally, the impact of different factors on students’ intentions is not static, and future prospective work can examine these trends over a long period of time. Online self-administered questionnaires introduce limitations, including sampling bias. To mitigate sampling bias, we followed the recruitment strategy outlined above and sent reminders to participants who had not completed the questionnaire. Lastly, our study provides a broad overview of the sample as a whole and should not be used to derive conclusions about specific institutions or individual cities in Saudi Arabia.

## Conclusions

The educational experience of medical students in Saudi Arabia is not uniform. Notably, female students and those from less urbanized hometowns reported less exposure to their preferred specialty. Limited educational opportunities have negative effects on students. Reduced exposure to a chosen specialty correlates with lower self-assessment scores and lower interest scores, highlighting the importance of broad exposure to medical specialties through diverse activities. It is crucial for future initiatives to address these disparities, ensuring that each student, irrespective of gender or hometown size, is provided with equal opportunities to explore their chosen specialty. This collective effort is necessary to cultivate a well-prepared medical workforce and address healthcare shortages, ensuring the equitable distribution of skilled medical professionals throughout the nation.
